# Supplementary material for: MetaRibo-Seq measures translation in microbiomes
Source: Nat Commun. 2020 Jun 29;11:3268. doi: 10.1038/s41467-020-17081-z (PMC7324362; doi:10.1038/s41467-020-17081-z)
Supplement: Supplementary file 10 — Supplementary Data 7 [file 41467_2020_17081_MOESM10_ESM.zip › File2/Confidence_High_Taxonomy/108126_out.krona.html]

Javascript must be enabled to view this page.

members
magnitude
magnitudeUnassigned
count
unassigned
taxon
rank

108126\_out

110

110
superkingdom
2

110
1239
phylum

110
186801
class

110
186802
order

104
31979
family


SRS024009\_contig\_number\_1133SRS1041091\_contig\_number\_contig-100\_16189.16190SRS140492\_contig\_number\_contig-100\_5024.88467SRS142503\_contig\_number\_contig-100\_3765.3765
genus
580596
104
4

1193532

SRS011134\_contig\_number\_contig-100\_3735.257730SRS011239\_contig\_number\_contig-100\_1584.158939SRS012969\_contig\_number\_33002SRS013965\_contig\_number\_40780SRS014979\_contig\_number\_7721SRS015960\_contig\_number\_contig-100\_5273.83077SRS016018\_contig\_number\_5253SRS016335\_contig\_number\_22353SRS017103\_contig\_number\_contig-100\_20069.145947SRS017821\_contig\_number\_contig-100\_4464.160896SRS018351\_contig\_number\_16218SRS018817\_contig\_number\_4732SRS019445\_contig\_number\_contig-100\_4030.51195SRS019693\_contig\_number\_1314SRS020271\_contig\_number\_3292SRS022609\_contig\_number\_49122SRS023526\_contig\_number\_23541SRS024132\_contig\_number\_49481SRS024663\_contig\_number\_contig-100\_3.93547SRS042628\_contig\_number\_34698SRS043701\_contig\_number\_12621SRS043841\_contig\_number\_contig-100\_3729.3730SRS044535\_contig\_number\_contig-100\_7861.52251SRS047014\_contig\_number\_17259SRS049773\_contig\_number\_18107SRS049995\_contig\_number\_36359SRS051610\_contig\_number\_9099SRS053356\_contig\_number\_46465SRS053573\_contig\_number\_3506SRS058070\_contig\_number\_31847SRS063985\_contig\_number\_18634SRS065504\_contig\_number\_contig-100\_463.215834SRS075341\_contig\_number\_6620SRS076804\_contig\_number\_contig-100\_1750.116787SRS076929\_contig\_number\_26860SRS077231\_contig\_number\_23039SRS077502\_contig\_number\_16567SRS077589\_contig\_number\_1038SRS077641\_contig\_number\_16064SRS077849\_contig\_number\_29917SRS098881\_contig\_number\_contig-100\_9711.101262SRS104084\_contig\_number\_7505SRS1041137\_contig\_number\_contig-100\_943.176364SRS1041145\_contig\_number\_39233SRS104311\_contig\_number\_34958SRS105082\_contig\_number\_18820SRS1054691\_contig\_number\_14379SRS142890\_contig\_number\_45259SRS143417\_contig\_number\_11341SRS143598\_contig\_number\_21823SRS143876\_contig\_number\_33503SRS144183\_contig\_number\_contig-100\_41.219002SRS144297\_contig\_number\_contig-100\_5430.5431SRS144506\_contig\_number\_23328SRS146764\_contig\_number\_22457SRS146812\_contig\_number\_36057SRS146813\_contig\_number\_contig-100\_294.157847SRS148319\_contig\_number\_17275SRS892882\_contig\_number\_2606SRS893358\_contig\_number\_9674SRS893373\_contig\_number\_17022SRS893378\_contig\_number\_15721SRS893383\_contig\_number\_contig-100\_90.179609SRS971275\_contig\_number\_45579
species
64

32
2292296

SRS013098\_contig\_number\_49969SRS013476\_contig\_number\_9775SRS013940\_contig\_number\_18420SRS014412\_contig\_number\_4331SRS014923\_contig\_number\_contig-100\_514.242250SRS015578\_contig\_number\_31423SRS015663\_contig\_number\_35300SRS016095\_contig\_number\_2113SRS017247\_contig\_number\_2401SRS018049\_contig\_number\_383SRS018936\_contig\_number\_contig-100\_14584.61682SRS021948\_contig\_number\_34724SRS049446\_contig\_number\_6479SRS049712\_contig\_number\_contig-100\_20298.89063SRS053335\_contig\_number\_contig-100\_8339.80702SRS055017\_contig\_number\_1102SRS057480\_contig\_number\_contig-100\_487.75330SRS065397\_contig\_number\_contig-100\_11724.11725SRS075078\_contig\_number\_30035SRS075773\_contig\_number\_27423SRS077392\_contig\_number\_38622SRS078419\_contig\_number\_37614SRS098073\_contig\_number\_16071SRS1041134\_contig\_number\_contig-100\_3378.3379SRS142599\_contig\_number\_5456SRS143523\_contig\_number\_contig-100\_16254.101449SRS143895\_contig\_number\_4994SRS147557\_contig\_number\_contig-100\_24304.71442SRS147614\_contig\_number\_contig-100\_10913.10914SRS148196\_contig\_number\_contig-100\_13410.164968SRS149325\_contig\_number\_14305SRS893279\_contig\_number\_2850
species

4
2292004

SRS024075\_contig\_number\_16668SRS050752\_contig\_number\_30394SRS050925\_contig\_number\_6002SRS051031\_contig\_number\_12752
species

1

SRS017916\_contig\_number\_11142
species
1898207

1
1392389
genus

species

SRS024331\_contig\_number\_contig-100\_3366.41959
1673721
1

family
216572
3

3
genus
459786

3
species

SRS050941\_contig\_number\_16140SRS074964\_contig\_number\_6834SRS142980\_contig\_number\_1645
1897011

family
541000
1

genus
946234
1

1

SRS056273\_contig\_number\_18349
292800
species
